# Corticotropin releasing factor-overexpressing mouse is a model of chronic stress-induced muscle atrophy

**DOI:** 10.1371/journal.pone.0229048

**Published:** 2020-02-12

**Authors:** Wesuk Kang, Tao Tong, Taesun Park

**Affiliations:** Department of Food and Nutrition, Brain Korea 21 PLUS Project, Yonsei University, Seodaemun-gu, Seoul, Korea; Max Delbruck Centrum fur Molekulare Medizin Berlin Buch, GERMANY

## Abstract

Chronic stress and continually high glucocorticoid levels can induce muscle atrophy. Unfortunately, there is a lack of appropriate animal models for stress-induced muscle atrophy research. Corticotropin releasing factor-overexpressing (CRF-OE) mice are a transgenic model of chronic stress that exhibit increased plasma corticosterone levels and Cushing’s syndrome; however, the skeletal muscle pathology of the CRF-OE mouse has not been well studied. We observed that male, 19-week-old CRF-OE mice had significantly lower skeletal muscle mass, average cross-sectional myofiber area, and total muscle protein content than their wild type (WT) littermates. Muscle function determined by grip strength, wire-hang, and open field tests showed that 19-week-old male CRF-OE mice had impaired physical ability. Additionally, the skeletal muscles of CRF-mice exhibited decreased expression of factors involved in the IGF-1/AKT/mTOR protein synthesis pathway and increased ubiquitin proteasome pathway activity compared to the WT control mice. In conclusion, 19-week-old CRF-OE mice display numerous features of muscle atrophy and thus serve as a model for investigating stress-induced muscle atrophy and interventions to target the deleterious effects of stress on skeletal muscle.

## Introduction

Skeletal muscle atrophy is characterized by a loss of muscle mass and function and can be a consequence of many noncommunicable and communicable diseases, for example cancers, sepsis, acquired immune deficiency syndrome (AIDS), respiratory diseases, and burn injury [1, 2]. Recently, lifestyle factors such as stress, low physical activity [3], poor diet [4, 5], alcohol abuse [6], and smoking [7] have also been linked to muscle atrophy. In particular, several lines of evidence have suggested that stressful life events are associated with muscle atrophy in both young and old individuals [8–10]. A separate study revealed that a single intravenous infusion of stress hormones (combination of cortisol, epinephrine, and glucagon) decreased skeletal muscle protein synthesis, as evidenced by the size distribution and concentration of ribosomes in healthy volunteers [11]. In rodents, combined acoustic and restraint stress for 5 days significantly decreased lean body mass [12], while restraint or cage-switching stress for 7 days induced the expression of atrophic genes such as myostatin and the loss of tibialis anterior muscle mass [13]. Similarly, exposing rats to 28-day restraint stress induced atrophy and apoptosis in the gastrocnemius muscle and significantly attenuated the phosphoinositide 3-kinase (PI3K)/ protein kinase B (AKT) pathway in the protein synthesis signaling [14].

Muscle atrophy essentially reflects an imbalance between a decreased synthesis and an increased breakdown of myofibrillary proteins. Thus, a variety of metabolic abnormalities, including mitochondrial dysfunction, androgen deficiency, growth hormone deficiency, insulin resistance and vitamin D deficiency has frequently been associated with muscle atrophy [15–17]. It is widely accepted that the hypothalamic-pituitary-adrenal (HPA) axis is a component of stress response and has its own intrinsic control points for regulating metabolic processes. Stress triggers the secretion of corticotropin releasing factor (CRF) from the hypothalamus, which activates the anterior pituitary to stimulate the synthesis and secretion of adrenocorticotropic hormone (ACTH). ACTH then stimulates the adrenal cortex to produce glucocorticoids [18, 19]. In response to acute stress, glucocorticoid output is modulated via negative feedback and activates inhibitory pathways that further reduce the HPA axis activity. On the other hands, chronic stress, which can occur via repeated exposure to stress, disrupts the glucocorticoid negative feedback system and leads to sustained high glucocorticoid levels [20]. Subsequently, glucocorticoids directly induce catabolism and decrease the rate of protein synthesis in skeletal muscle, resulting in a profound loss of muscle mass [21, 22].

In an experimental stress model, the research purpose must be clearly defined and a variety of factors that may change individual aspects of the chronic stress response must be considered. Repeated exposure to a variety of stressors has been reported to lead to a gradual attenuation of the response of stress-induced plasma corticosterone production in rodents, termed habituation [23–25]. In 1992, Stenzel-Poore et al. developed corticotropin-releasing factor overexpressing (CRF-OE) transgenic mice to investigate the physiological consequences of CRF overproduction leading to Cushing-like syndromes [26]. Besides truncal obesity and bilateral symmetric hair loss, they reported these transgenic mice displayed muscle wasting as evidenced by appearance. These mice also display elevated plasma corticosterone levels associated with chronic activation of the HPA axis alongside anxiogenic behaviors [27], brain atrophy [28], and exaggerated voiding and defecation in response to novel environmental stressors [29]. Furthermore, CRF-OE mice recapitulated many features of osteoporosis with increased expression of muscle atrophy gene markers [30]. In the present study, we aimed to assess muscle atrophy in the CRF-OE mouse model, and elucidate its molecular mechanism.

## Materials and methods

### Animals

CRF-OE mice (hemizygous) originally developed by Vale *et al* [26] (in which rat CRF is expressed under the control of the mouse metallothionein promoter) were purchased from Jackson Labs (Bar Harbor, ME, USA). Male CRF-OE mice were bred for 3–4 generations with female C57Bl/6 mice to generate sufficient CRF mice and corresponding wildtype (WT) littermates. For the experiment, five-week-old male and female CRF-OE mice and their sex- and age-matched WT littermates, as control groups, were housed in standard cages in a room at 22 ± 2.0°C and 50 ± 5% relative humidity and maintained under 12 h:12 h light-dark conditions with a commercial diet (AIN 93 M, Purina Mills, St. Louis MO, USA) and distilled water *ad libitum*. It was previously reported that CRF-OE mice showed various pathological changes from 6 to 21 weeks of age (eg, osteoporosis, insulin resistance, hypercortisolism and obesity) [30–33]; We chose to use CRF-OE mice at 7 and 19 weeks of age to investigate muscle atrophy. All experimental procedures were reviewed and approved by the Institutional Animal Care and Use Committee of the Yonsei Laboratory Animal Research Center (Permit no. IACUC-A-201704-188-01).

### Blood and tissue sample collection

Twice during the experimental period (7 and 19 weeks old, n = 7 in each group of each sex), mice were intraperitoneally anesthetized with Avertin (200 mg/kg, Sigma-Aldrich) and euthanized by exsanguination via carotid artery between 9:00 and 11:00 hours following a 6 h fast and all efforts were made to minimize suffering. Blood (300–400 μL) was collected from the mice via retro-orbital puncture using heparinized capillary tubes and plasma was stored at −80°C until further use. The brain, adrenal glands, muscles (rectus femoris, gastrocnemius, and soleus), and visceral fat (epididymal, perirenal, mesenteric, and retroperitoneal) were removed, weighed, and immediately stored at –80°C until further analysis.

### Biochemical analyses

Plasma glucose concentration was measured using commercial kits (V-glucose, Asan Pharmaceutical, Korea). Plasma corticosterone, monocyte chemoattractant protein-1 (MCP1), interleukin 6 (IL-6), and insulin levels were determined using commercially available enzyme‐linked immunosorbent assay (ELISA) kits (Millipore, MA, USA). All procedures were conducted according to the manufacturer's instructions. To assess insulin resistance, homeostasis model assessment of basal insulin resistance (HOMA-IR) was calculated as follows:

$$\frac{Fasting\;plasma\;glucose\times fasting\;plasma\;insulin}{22.5}.$$

To analyze muscle protein content, rectus femoris, gastrocnemius, and soleus muscle samples were homogenized in 40 volumes of a lysis buffer containing 100 mmol/L Tris-HCl (pH 7.4), 100 mmol/L orthovanadate, 50 mmol/L sodium pyrophosphate, 50 mmol/L NaF, 50 mmol/L NaCl, 5 mmol/L EDTA, 1 mmol/L phenylmethanesulfonyl fluoride, 1% Triton X-100, 2 μg/mL aprotinin, 1 μg/mL leupeptin, and 1 μg/mL pepstatin A. The samples were centrifuged at 13,000 × *g* for 20 min at 4°C and the protein content of the homogenate was measured using a Bio-Rad protein assay (Bio-Rad, Hemel Hempstead, UK) with bovine serum albumin as the protein standard.

### Grip strength and wire hanging measurements

Four-limb grip strength was measured using an automated grip-strength meter (Daejong Instrument Industry, Seoul, Korea). The mouse was allowed to grasp a metal pull bar with all four paws. Its tail was then gradually pulled backward horizontally until the mouse lost its grip. The force applied to the bar at the moment grasp was released was recorded as the peak tension. Each mouse was tested five times with a 30–50 s period between two successive trials; the average peak tension and maximum peak tension were normalized to body weight to represent four-limb grip strength. For the hanging wire test, mice were placed on wire mesh and made to grasp the wire using a commercially available rotarod apparatus (Daejong Instrument Industry, Seoul, Korea). The wire net was turned upside down and the time taken to fall off was measured. Each mouse was tested five times with a 2–3 min period between two successive trials; the average time and maximum time to fall were normalized to body weight to represent fall latency.

### Locomotion assessment

Individual mice were placed in 50 × 50 cm acrylic movement boxes. Their spontaneous locomotion was measured using a camera positioned above the boxes. Activity was recorded for 10 minutes and estimated using an automated video-tracking system (Harvard Apparatus, Holliston, MA, USA). The number of movements and distance moved (in cm) were analyzed and averaged for each mouse.

### Histological analysis

To assess muscle morphology and quantify the cross-sectional area (CSA) of muscle fibers, 5 μm-thick sections of the soleus, rectus femoris, and gastrocnemius muscles were stained with hematoxylin and eosin (H&E) and examined using an Olympus IX71 microscope equipped with a DP-70 camera (Olympus, Center Valley, PA, USA). Fiber CSA was calculated by analyzing ∼250 myofibers using ImageJ software (Java software; National Institute of Health).

### Total RNA extraction and qRT-PCR

Gastrocnemius muscle samples were homogenized and total RNA was extracted using TRIzol® reagent (Invitrogen, Carlsbad, CA, USA) according to the manufacturer's instructions. Total RNA (4 μg) was reverse transcribed (RT) to complementary DNA (cDNA) using a RevertAidTM first strand cDNA synthesis kit (Fermentas, Maryland, USA) in a 40 μL reaction mixture. qRT-PCR was conducted and monitored using an ABI Prism ® 7000 sequence detection system (Applied Biosystems, Foster City, CA, USA) with 10 μL of SYBR Green reagent (Takara Bio, Shiga, Japan), 1 μL of each primer (25 μM), and 3 μL of cDNA. Samples were run in triplicate and relative mRNA expression was measured by cycle threshold (CT) values. All primers used for qRT-PCR are shown in Table 1. Gene expression data were normalized against GAPDH levels.

**Table 1:** Primer sequences.

Gene description	Sequences (5’→3)
*Atrogin-1*	F: GTCCAGAGAGTCGGCAAGTC
	R: GTCGGTGATCGTGAGACCTT
*Muscle RING finger 1* (*MuRF1*)	F: ACATCTACTGTCTCACGTGT
	R: TGTCCTTGGAAGATGCTTTG
*Myostatin*	F: TCACGCTACCACGGAAACAA
	R: AGGAGTCTTGACGGGTCTGA
*Insulin-like growth factor-1* (*IGF-1*)	F: TGCTGATTTTCCCCATCGCT
	R: AGAGCCTGCGCAATGGAATA
*11β-Hydroxysteroid dehydrogenase* (*11β-HSD*)	F: GTGTCTCGCTGCCTTGAACTC
	R: TTTCCCGCCTTGACAATAAATT
*p85*	F: GAGGATTTGCCCCACCATGA
	R: CCACTACGGAGCAGGCATAG
*Kruppel-like factor 15* (*KLF15*)	F: CTGCAGCAAGATGTACACCAA
	R: TCATCTGAGCGTGAAAACCTC
*Regulated in development and DNA damage response-1* (*REDD-1*)	F: CCAGAGAAGAGGGCCTTGA
	R: CCATCCAGGTATGAGGAGTCTT
*Glyceraldehyde-3-phosphate dehydrogenase* (*GAPDH*)	F: GTGATGGCATGGACTGTGGT
	R: GGAGCCAAAAGGGTCATCAT
Myh1	F: AGCTTCAAGTTTGGACCCACG
	R: TTGGTTGCAGCCCAGTGAGA
Myh2	F: AAAGCTCCAAGGACCCTCTTATTTC
	R: AGCTCATGACTGCTGAACTCAC
Myh4	F: TTCCTATTTTCTGGGGGACAAGC
	R: AAATTCTCCCTGAAGAGAGCTGAC
Myh7	F: AACCTGTCCAAGTTCCGCAAG
	R: TTGGAGCTGGGTAGCACAAG

### Western blot analysis

The presence of various proteins was quantitated by performing western blot analysis. The gastrocnemius muscles and soleus of the mice were homogenized in the above-mentioned lysis buffer. Approximately 30 μg of protein was added to each lane of a 10% SDS-polyacrylamide gel and transferred onto a nitrocellulose membrane (Amersham Biosciences, Piscataway, NJ, USA). The membranes were blocked with 5% bovine serum albumin at room temperature for 1 h and incubated with primary antibodies at 4°C overnight, followed by the corresponding secondary antibodies. All primary antibodies were sourced from Cell Signaling Technology (Danvers, MA, USA) and horseradish peroxidase–conjugated anti-rabbit IgG antibodies purchased from Santa Cruz Biotechnology (Santa Cruz, CA, USA) were used as secondary antibodies. ECL Western blotting detection reagent (GE Healthcare, Little Chalfont, UK) was used to image the protein bands which were visualized on a WSE-6100 LuminoGraph system (ATTO, Tokyo, Japan) and quantified using the Quantity One program (Bio-Rad).

### Statistical analysis

Results are expressed as the mean ± s.e.m. Unpaired Student *t*-tests were used to analyze all data comparisons between the CRF-overexpression mice and WT littermate controls. All statistical analyses were performed using SPSS statistical software (SPSS Inc., Chicago, Il., USA) with significance set at *P < 0.05, **P < 0.01, and ***P < 0.001.

## Results

### Physiological characteristic alterations observed in CRF-OE mice

**Fig 1:**
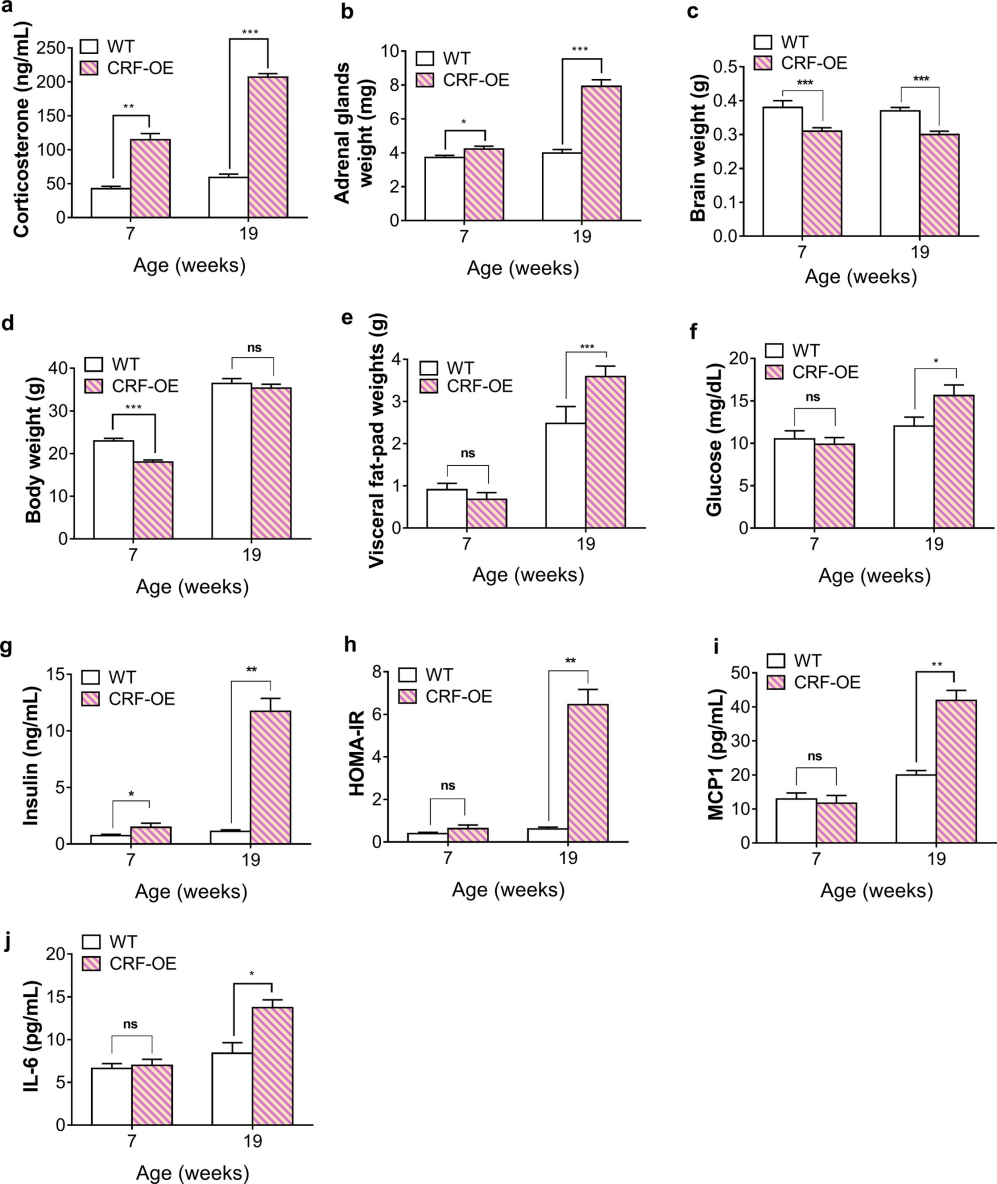
Physiological characteristic alterations observed in CRF-OE mice. (a) Plasma corticosterone concentration. (b) Adrenal gland weight. (c) Brain weight. (d) Body weight. (e) Visceral fat-pad weight. Plasma (f) glucose and (g) insulin levels. (h) HOMA-IR. Plasma (i) MCP1 and (j) IL-6 levels. *P < 0.05, **P < 0.01, and ***P < 0.001 compared to WT mice. Values represent the mean ± s.e.m (n = 7).

We followed the development of characteristics in CRF-OE mice at 7 and 19 weeks by measuring several physiological parameters. CRF-OE mice demonstrated a significant increase in plasma corticosterone levels at both ages compared to the age-matched control mice (Fig 1A). Moreover, the adrenal glands of CRF-OE mice were significantly heavier than those of the controls at both ages. In contrast, CRF-OE mice had significantly lower brain weights than their WT controls at both ages. Although the two genotypes had similar body weights at 19 weeks, the CRF-OE mice had higher visceral fat-pad weights than the age-matched WT mice (Fig 1D and 1E). Insulin resistance (calculated via HOMA-IR, an index derived from fasting glucose and insulin levels) was observed in the CRF-OE mice at 19 weeks but not at 7 weeks (Fig 1F, 1G and 1H). Similarly, the circulating levels of MCP1 and IL-6, two well-known adipokines, were significantly higher than those in the WT control mice at 19 weeks, but the differences were not significant at 7 weeks (Fig 1I and 1J).

### CRF-OE mice displayed muscle weakness

**Fig 2:**
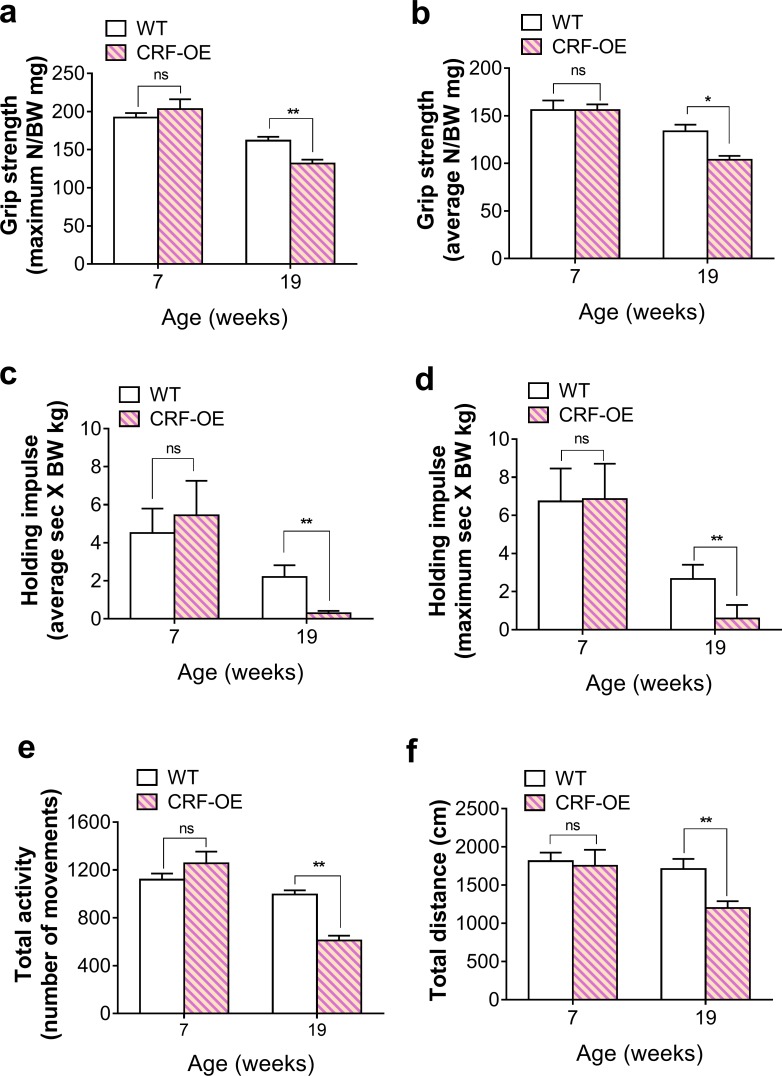
CRF-OE mice displayed muscle weakness. (a and b) Four-limb grip strength and (c and d) wire hanging fall latency normalized to body weight. (e) Number of movements. (f) Total distance travelled. *P < 0.05 and **P < 0.01 compared to WT mice. Values represent the mean ± s.e.m (n = 7).

The muscle function of CRF-OE mice was assessed by grip strength, wire hang, and open field tests. At 7 weeks, there were no significant differences in any of the test results between the two genotypes. In contrast, four-limb grip strength (male: Fig 2A and 2B; female: supplementary Fig 1A and 1B) and wire hang fall latency (male: Fig 2C and 2D; female: S1C and S1D Fig) were significantly lower in the CRF-OE mice than in the WT controls at 19 weeks. Furthermore, the number of movements and total distance travelled in the open-field test were also significantly lower for the 19-week-old male CRF-OE mice than for the age-matched WT controls (Fig 2E–2F).

### CRF-OE mice exhibited decreased muscle mass and fiber size

**Fig 3:**
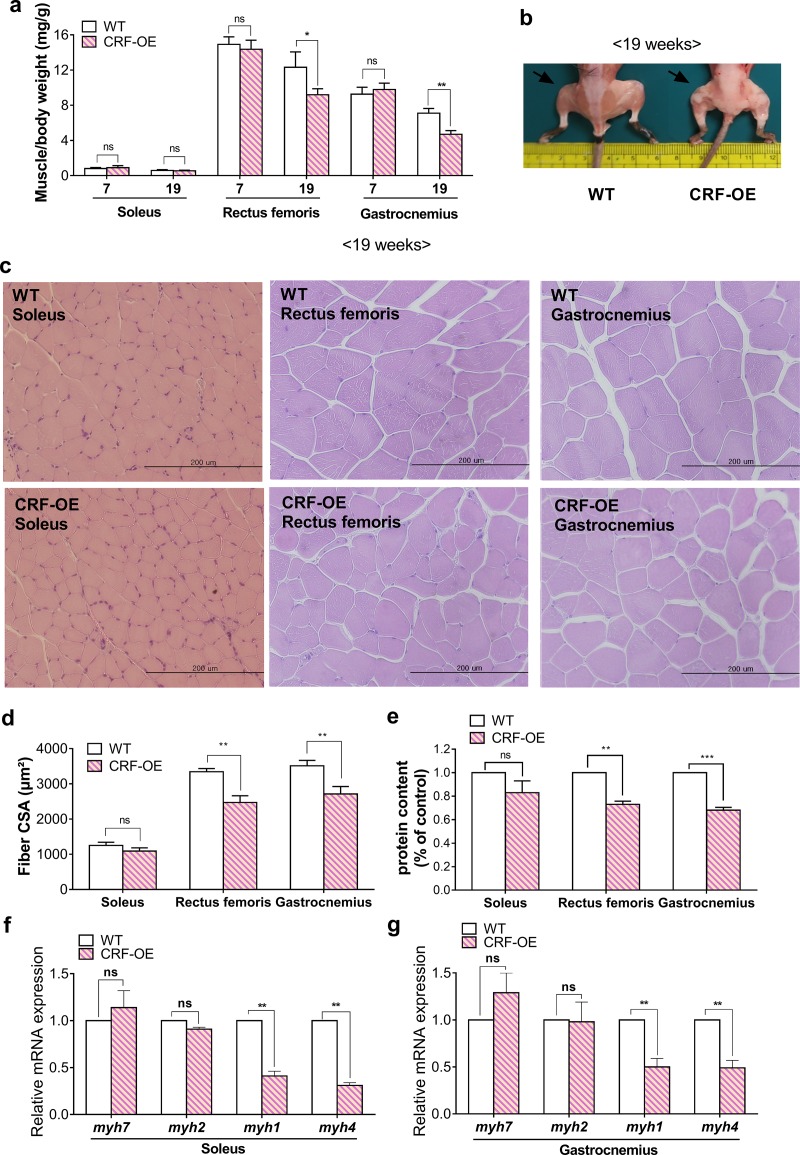
CRF-OE mice exhibited decreased muscle mass and fiber size. (a) Soleus, rectus femoris, and gastrocnemius muscle weights. (b) Image of mouse hindlimbs at 19 weeks. (c) Representative H&E-stained sections, (d) fiber average cross-sectional area (CSA), and (e) total cellular protein of soleus, rectus femoris, and gastrocnemius muscles at 19 weeks. qRT–PCR analysis of mRNA expression of myosin heavy chain genes in (f) soleus and (g) gastrocnemius muscles at 19 weeks. *P < 0.05, **P < 0.01, and ***P < 0.001 compared to WT mice. Values represent the mean ± s.e.m (n = 7).

We investigated whether the muscle dysfunction observed in CRF-mice was reflected in skeletal muscle mass and histopathology. At 19 weeks, rectus femoris and gastrocnemius muscle weights were significantly lower in CRF-OE mice than in the WT controls, whereas the soleus muscle was not significantly affected (male: Fig 3A and 3B; female: S1E Fig). Since both sexes were found to be almost equally susceptible to muscle atrophy induced by CRF-overexpression as evidenced by decreased muscle mass and function, all subsequent analyses of muscle tissues were conducted in male mice only. The CSA and total cellular protein content were also significantly lower in the rectus femoris and gastrocnemius muscles of male CRF-OE mice than in the WT controls, but not in the soleus muscles (Fig 3C, 3D and 3E). No significant differences were observed in the weights of all three muscles between 7-week-old CRF-OE mice and the age-matched WT controls. Furthermore, levels of mRNAs representing fast fiber types (Myh1 and Myh4) but not slow and intermediate fiber type (*Myh7 and Myh2*) were significantly decreased in the gastrocnemius and soleus muscles of CRF-OE mice compared to those of WT control mice (Fig 3F and 3G). Glucocorticoid receptor (GR) levels of gastrocnemius muscles were higher than those of soleus muscles in CRF-OE mice (S2 Fig).

### Reduced protein synthesis and stimulated breakdown signaling in CRF-OE mice

**Fig 4:**
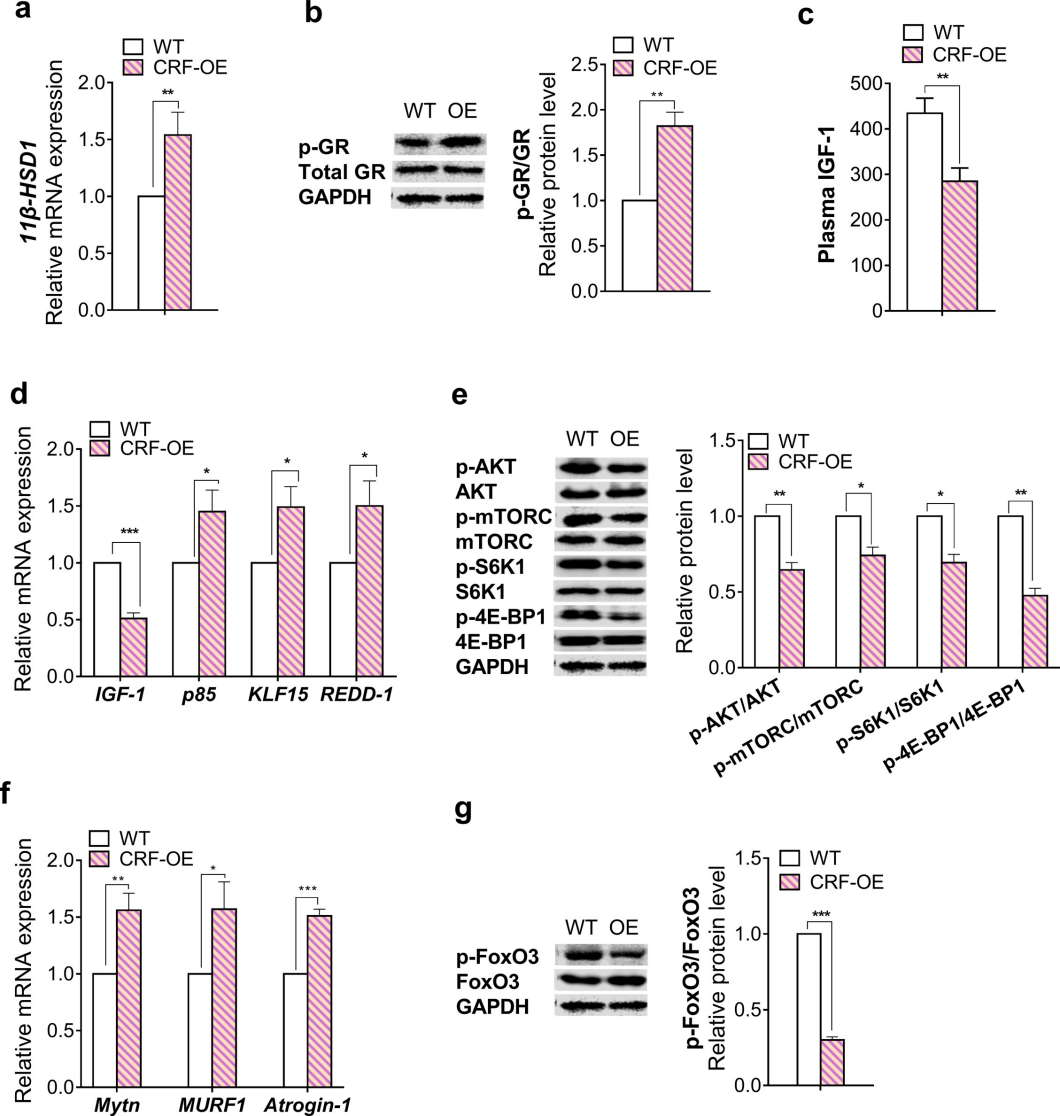
Reduced protein synthesis and stimulated breakdown signaling were observed in CRF-OE mice. (a) qRT–PCR analysis of relative 11beta-HSD1 mRNA expression. (b) Western blot analysis of glucocorticoid receptor (GR) phosphorylation. (c) Plasma IGF-1 concentration. mRNA and protein expression related to muscle protein synthesis were examined by (d) qRT–PCR and (e) western blot analysis, respectively. mRNA and protein expression related to muscle protein breakdown were examined by (f) qRT–PCR and (g) western blot analysis, respectively. *P < 0.05, **P < 0.01, and ***P < 0.001 compared to WT mice. Values represent the mean ± s.e.m. of three experiments. In each experiment, sample was pooled from 5–6 mice in each group.

**Fig 5:**
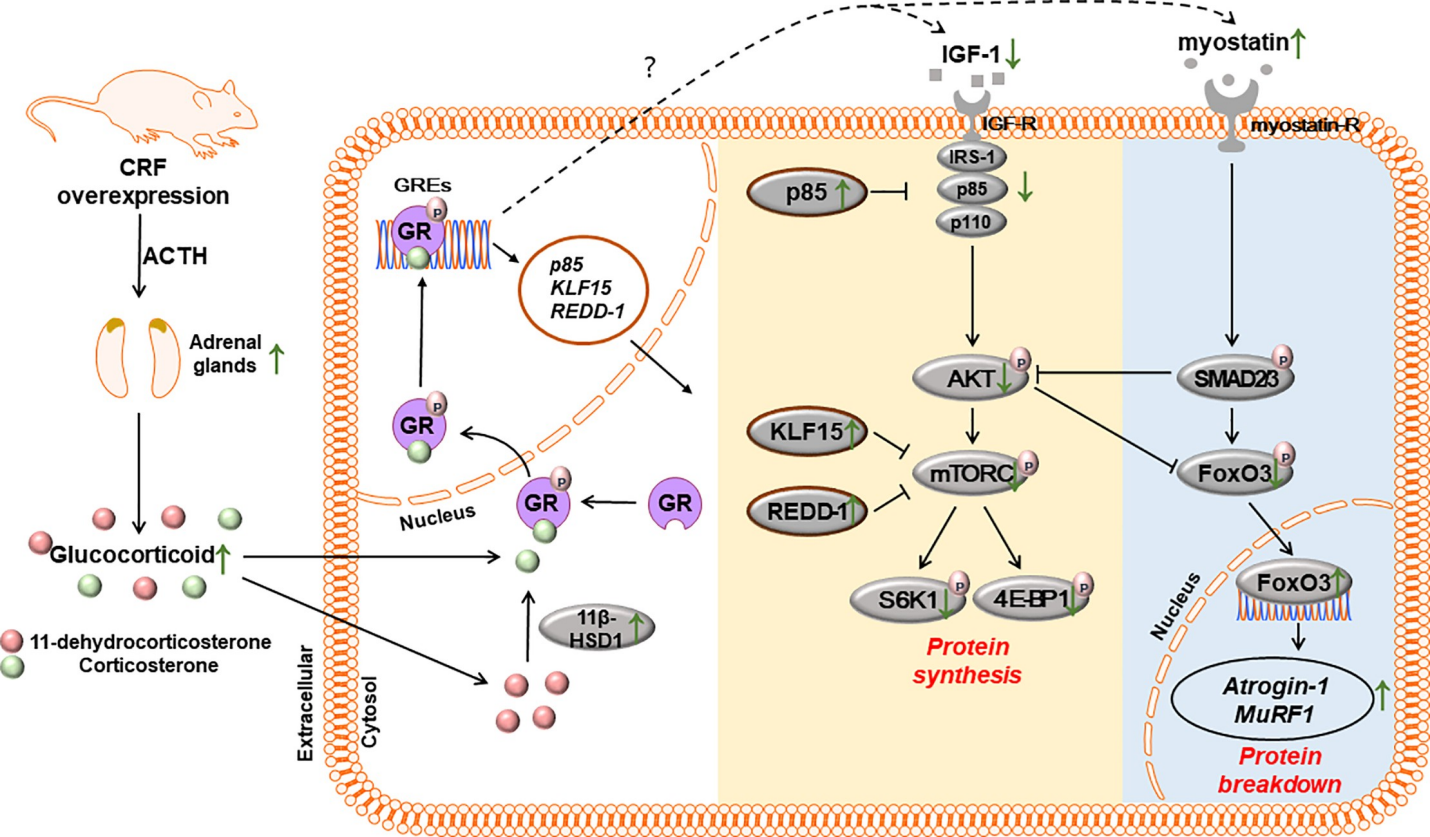
Schematic diagram illustrating the proposed mechanism of muscle atrophy in CRF-OE mice.

The gastrocnemius muscles of 19-week-old male mice were used to investigate possible mechanisms contributing to the muscle atrophy observed in CRF-OE mice. The *11β-Hydroxysteroid dehydrogenase* (*11-HSD1)* mRNA level was significantly higher in the gastrocnemius of CRF-OE mice than in the WT controls (Fig 4A). The GR maintains basal phosphorylation levels and is hyperphosphorylated upon binding glucocorticoid molecules. The skeletal muscles of CRF-OE mice exhibited significantly higher GR phosphorylation levels than the WT controls (Fig 4B). Regarding protein synthesis, both plasma Insulin-like growth factor-1 (IGF-1) and muscle *IGF-1* mRNA levels were significantly lower in CRF-OE mice than in the WT controls (Fig 4B and 4C). In addition, the mRNA levels of *p85*, *Kruppel-like factor 15 (KLF15)*, and *Regulated in development and DNA damage response-1* (*REDD-1*), direct GR target genes, were significantly higher in the muscles of CRF-OE mice than in the WT controls (Fig 4C). The phosphorylation levels of AKT, mammalian target of rapamycin complex 1 (mTORC1), S6 kinase 1 (S6K1), and eukaryotic initiation factor 4E-binding protein 1 (4E-BP1) were significantly lower in the muscles of CRF-OE mice than those in the WT controls (Fig 4D). Regarding protein degradation, CRF-OE mice exhibited upregulated *myostatin* mRNA expression and decreased forkhead box O3 (FoxO3) phosphorylation in the skeletal muscles compared with the WT controls (Fig 4E and 4F). The mRNA expression of *atrogin-1* and *Muscle RING finger 1* (*MuRF1)*, two transcriptional targets of FoxO3 involved in the ubiquitin–proteasome pathway, was also higher in the muscles of CRF-OE mice than in the WT controls (Fig 4F). Taken together, glucocorticoid-induced inhibition of IGF-1/AKT/mTOR protein synthesis pathway and activation of the ubiquitin–proteasome pathway are likely mechanism contributing to muscle atrophy in CRF-OE mice. (Fig 5).

## Discussion

Skeletal muscle can be divided into slow-twitch oxidative muscle (i.e. soleus) and fast-twitch glycolytic myofibers (i.e. gastrocnemius, rectus femoris, and tibialis anterior) that respond differently to exogenous or endogenous stimuli. Fast-twitch, glycolytic muscles are known to be more vulnerable to glucocorticoid-induced muscle atrophy than slow-twitch oxidative muscles [34, 35]. In a glucocorticoid-induced rat atrophy model (intraperitoneal administration for 5 days), treatment with dexamethasone significantly reduced the gastrocnemius weight as well as its myofiber CSA without significantly altering the soleus weight and its myofiber CSA [36]. The mechanism of muscle type specificity was suggested to be related to the higher level of GRs in the gastrocnemius than in the soleus muscles, in line with our results [37]. Consistently, we observed a CRF overexpression-induced reduction in muscle weight and myofiber CSA in the gastrocnemius and rectus femoris muscles, but not in the soleus.

Over the past few decades, the importance of maintaining muscle strength and mass has motivated extensive research into muscle growth pathways. These studies have emphasized the importance of the IGF-1/PI3K/AKT pathway in boosting protein synthesis by generally activating translation via S6K1 and 4E-BP1 phosphorylation (two factors that play a role in protein synthesis machinery by controlling the initiation step of mRNA translation) and by inhibiting genes involved in ubiquitin-proteasome protein degradation, such as *atrogin-1* and *MURF1* [38]. In contrast, myostatin, also known as growth and differentiation factor 8, has been identified and shown to negatively regulate muscle mass [39]. Myostatin overexpression can reduce muscle gene expression and negatively modulate AKT-mTOR signaling, whereas knocking out the myostatin gene in mice blunts the induction of FoxO3, atrogin-1, and MURF1 expression and prevents muscle wasting [40–42]. In the skeletal muscle, it has been demonstrated that GC-induced atrophy results mainly from the inhibition of IGF-1 signaling and activation of myostatin signaling. At the molecular level, cytosolic GR enters the nucleus upon binding to GCs and associates with glucocorticoid response elements (GREs), leading to the recruitment of transcription cofactors to activate or repress the transcriptional rate of nearby genes (such as *KLF15*, *REDD-1* and *p85*) known to modulate the IGF-1 and myostatin systems [43, 44]. In this study, the inactivation of IGF-1 signaling and activation of myostatin signaling were observed in gastrocnemius muscles of CRF-OE mice compared with those in the WT control group, coinciding with a significant increase in the plasma GC concentration and upregulation of its target genes.

Increased fat mass, particularly visceral adipose tissue, is known to induce chronic subclinical inflammation, closely affecting muscle mass and function. Adipokines generated by visceral fat pads, such as IL-6, are thought to influence growth hormone secretion and insulin resistance, which are strongly associated with muscle atrophy [45–47]. Alternatively, it has been suggested that elevated plasma IL-6 levels can directly cause muscle atrophy by activating proteolytic systems in muscle: IL-6/IL-6 receptor interaction leads to activation of signal transducer and activator of transcription (STAT) signaling pathways, which are known to inhibit muscle differentiation and regeneration [48–50]. In this study, increases in visceral adipose tissues and plasma IL-6 were observed in CRF-OE mice compared with that in the WT control group alongside a decrease in muscle mass and function. Our results suggest that the atrophic properties of CRF-OE mice may be partially attributed to increased visceral fat pad mass and circulating IL-6 level.

Accumulating data from transgenic mice has increased our understanding of the phenotypic effects of specific molecules. The use of a diverse array of measurements should be considered, since genetic modifications often lead to test-specific results; meanwhile, test standardization assures a higher degree of interlaboratory replicability [51, 52]. Consequently, we assessed skeletal muscle function using three well-documented tests: grip strength, wire-hang, and open field tests, which have been adopted as part of the comprehensive SHIRPA phenotypic testing regimen conceived as a multi-test battery with standardized guidelines and materials [53]. The grip strength test estimates muscle force and neuromuscular integration relating to the grasping reflex, whereas the four-limb wire hanging test measures muscle coordination and endurance in mice [54, 55]. Meanwhile, the open field activity test comprehensively assesses the behavioral and locomotor activity levels of rodents, which are positively associated with locomotive function [56]. In the present study, locomotor activity was impaired in CRF-OE mice at the age of 19 weeks. Given that CRF-OE mice exhibit anxiety-like behavior [27], we cannot exclude the possibility that anxiety also contributes to impaired locomotor performance in CRF-OE mice [56, 57].

Both 7- and 19-week-old CRF-OE mice exhibited a significant increase in plasma corticosterone levels compared with their WT littermates; however, this increase in plasma corticosterone levels was only paralleled by decreases in skeletal muscle mass and function at 19 weeks. One possible explanation for these results is that muscle atrophy may occur only when plasma corticosterone levels exceed a certain threshold. Further investigation is required to understand the relationship between corticosterone levels and its ability to induce muscle atrophy. Another point worth considering is the age of the animal at the time of exposure to elevated plasma corticosterone levels. In agreement with our results, it was reported that wasting was slower in glucocorticoid-treated young rats muscle than in aged rats and that muscle mass recovery took half as long as that in the aged rats [58]. In addition, dexamethasone treatment decreased hindlimb grip strength in both young and old rats, but the extent of decrease was significantly greater in the old rats [59].

In conclusion, our results indicate that 19-week-old CRF-OE mice display many features that can be attributed to muscle atrophy, including loss of muscle function, reduced muscle mass and fiber size, and decreased hypertrophy and increased atrophy markers at the gene and protein level. Overall, these data show that CRF-OE mice are a useful model for studying stress-induced muscle atrophy and testing new therapeutic interventions to mitigate muscle loss.

## Supporting information

S1 Fig Female CRF-OE mice exhibited decreased muscle function and mass. (a and b) Four-limb grip strength and (c and d) wire hanging fall latency normalized to body weight. (e) Soleus, rectus femoris, and gastrocnemius muscle weights. *P < 0.05 and **P < 0.01 compared to WT mice. Values represent the mean ± s.e.m (n = 7).
(TIF)

S2 Fig Glucocorticoid receptor (GR) levels of gastrocnemius muscles were higher than those of soleus muscles in CRF-OE mice. Western blot analysis of GR levels in soleus and gastrocnemius muscles of CRF-OE mice. Values represent the mean ± s.e.m. of three experiments. In each experiment, sample was pooled from 5–6 mice in each group.
(TIF)

S1 Raw images (PDF)
