# Supplementary figures and images for: Corticotropin releasing factor-overexpressing mouse is a model of chronic stress-induced muscle atrophy
Source: PLoS One. 2020 Feb 12;15(2):e0229048. doi: 10.1371/journal.pone.0229048 (PMC7015416; doi:10.1371/journal.pone.0229048)

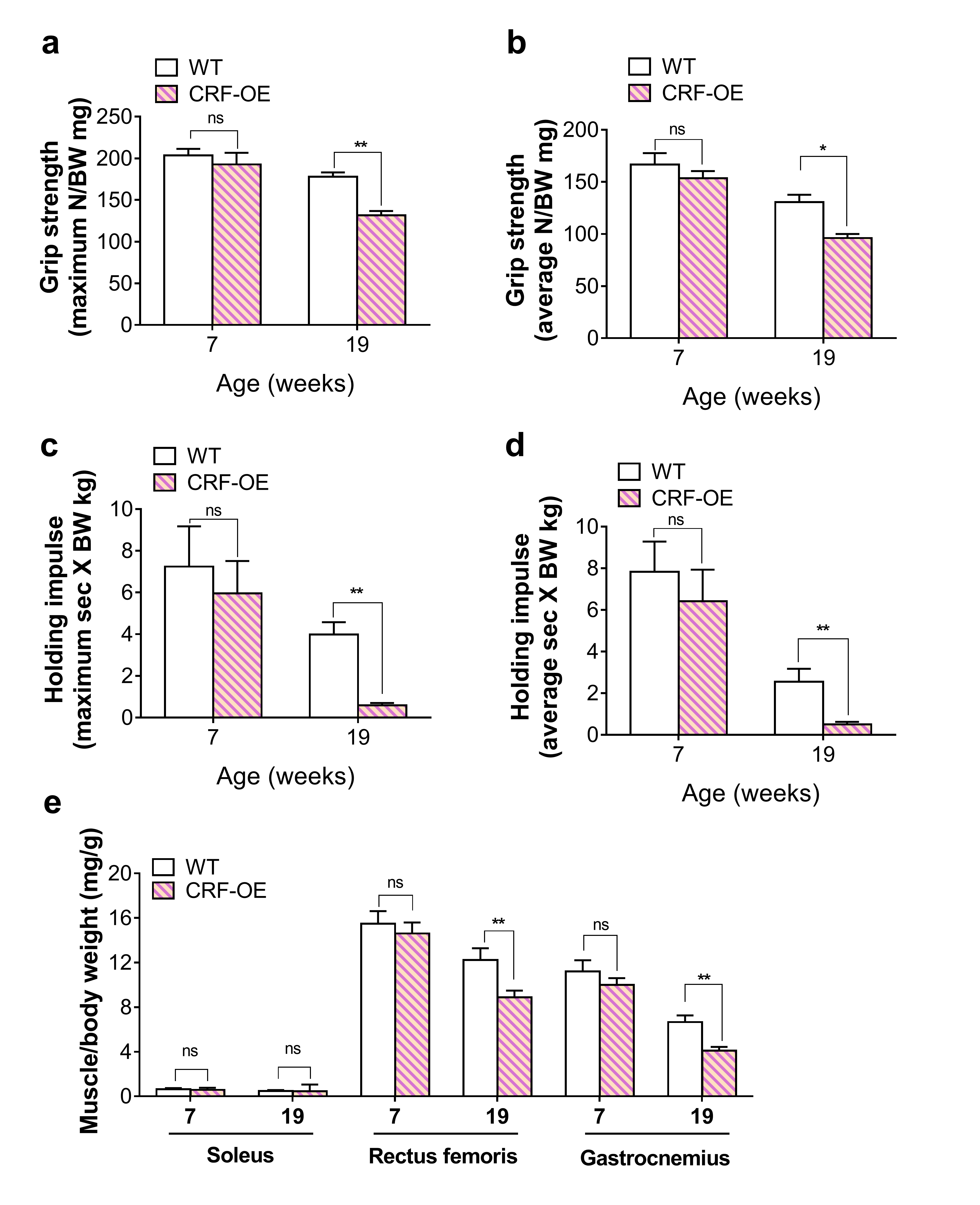

Supplement: S1 Fig — (a and b) Four-limb grip strength and (c and d) wire hanging fall latency normalized to body weight. (e) Soleus, rectus femoris, and gastrocnemius muscle weights. *P < 0.05 and **P < 0.01 compared to WT mice. Values represent the mean ± s.e.m (n = 7). (TIF) [file pone.0229048.s001.tif]

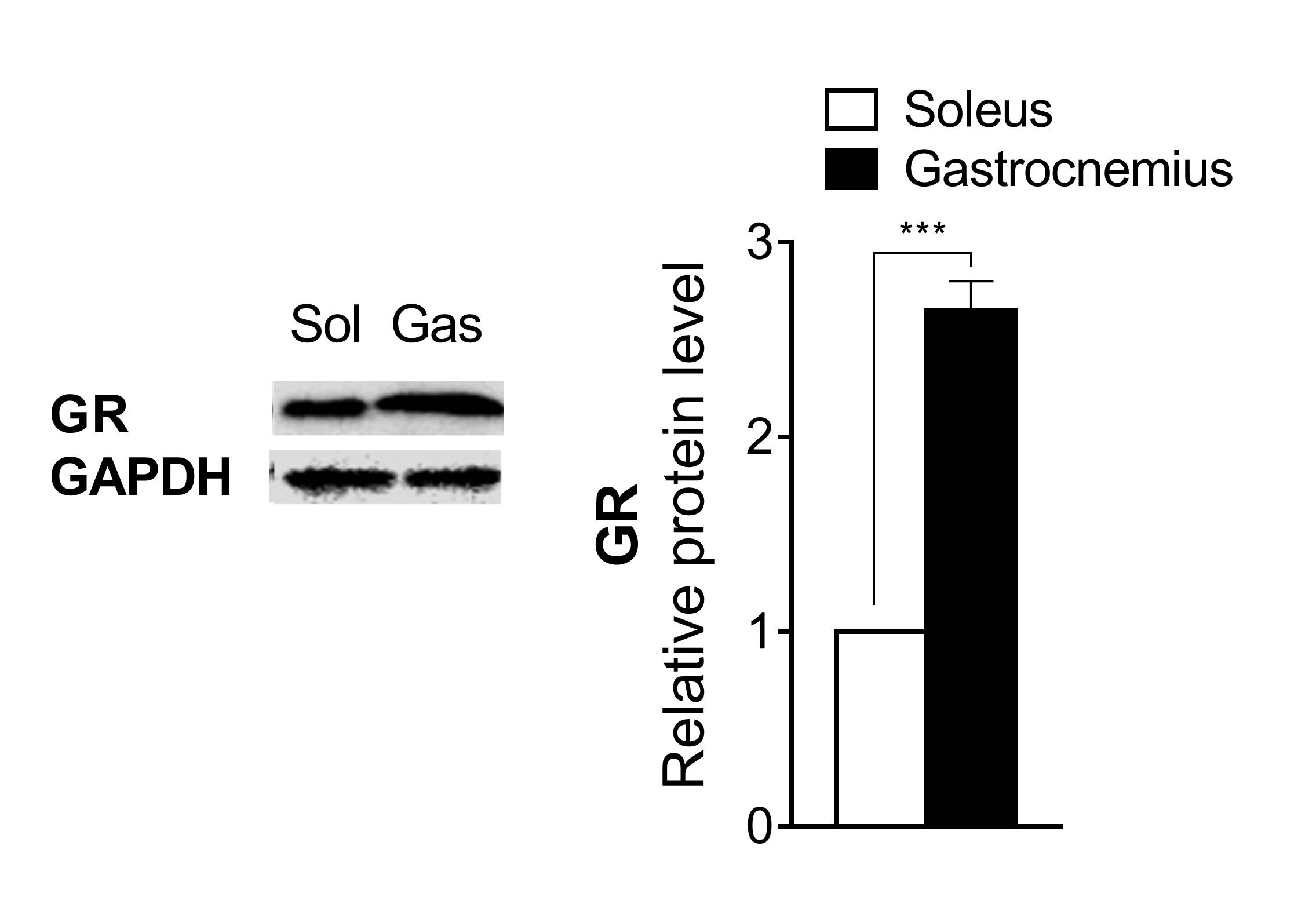

Supplement: S2 Fig — Western blot analysis of GR levels in soleus and gastrocnemius muscles of CRF-OE mice. Values represent the mean ± s.e.m. of three experiments. In each experiment, sample was pooled from 5–6 mice in each group. (TIF) [file pone.0229048.s002.tif]

Fig. 4b.

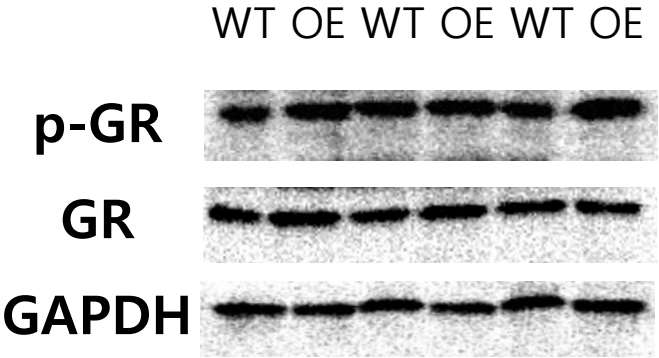

Fig. 4e.

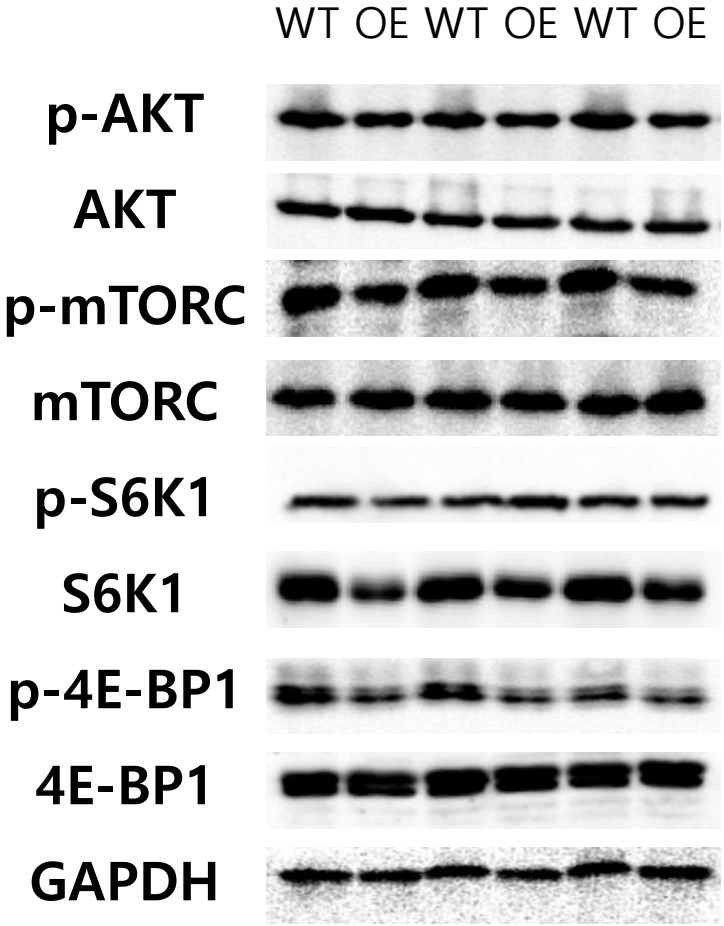

Fig. 4g.

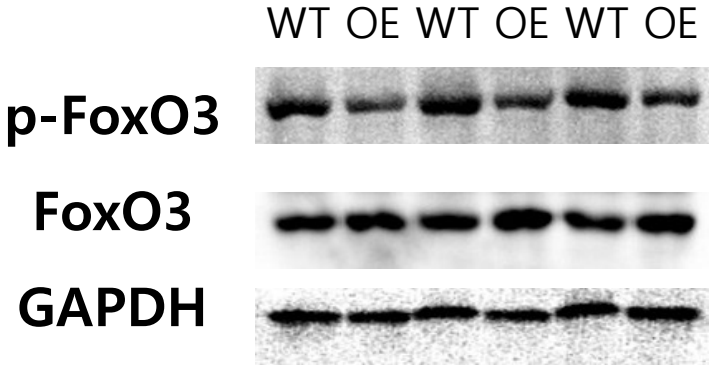

Supplementary Fig. 2.

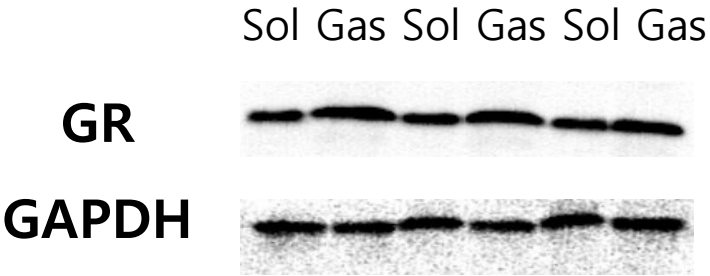

Supplement: S1 Raw images — (PDF) [file pone.0229048.s003.pdf]
